# Observation of Palatal Wound Healing Process Following Various Degrees of Mucoperiosteal and Bone Trauma in a Young Rat Model

**DOI:** 10.3390/biology11081142

**Published:** 2022-07-29

**Authors:** Yingmeng Liu, Shiming Zhang, Karim Ahmed Sakran, Jiayi Yin, Min Lan, Chao Yang, Yan Wang, Ni Zeng, Hanyao Huang, Bing Shi

**Affiliations:** State Key Laboratory of Oral Diseases, National Clinical Research Center for Oral Diseases, Department of Oral Maxillofacial Surgery, West China Hospital of Stomatology, Sichuan University, Chengdu 610041, China; liuyingmeng_cn@163.com (Y.L.); shimmy_zhang@163.com (S.Z.); sakrandent89@gmail.com (K.A.S.); scuyjy@sina.com (J.Y.); lanmin1109@163.com (M.L.); 28611736@163.com (C.Y.); wangyancn2022@163.com (Y.W.); shibingcn@vip.sina.com (B.S.)

**Keywords:** palatal wound healing, acellular dermal matrix, maxillary growth, mucoperiosteal trauma

## Abstract

**Simple Summary:**

The exact correlation of palatal trauma to maxillary inhibition has not been demonstrated. This paper determines the influence of different degrees of palatal trauma on maxillofacial growth and assesses whether usage of ADM can help rescue the inhibited growth during palatal wound healing. This research would help the surgeons comprehensively understand the impact of palatal trauma on maxillary growth and the therapeutic effect of the ADM.

**Abstract:**

The accidental injury or surgery on soft and hard palatal tissue has an adverse impact on normal maxillary morphology. To design a single-factor experiment that excludes other interfering factors on maxillary growth, a young rat model was established to simulate the various degrees of palatal trauma. Eight maxillary parameters were measured to evaluate the impact of palatal trauma on maxillary growth. Furthermore, the acellular dermal matrix (ADM) was applied to cure the palatal trauma and alleviate the adverse impact of bone denudation on the maxillary growth. Micro-CT scanning and histology analyses were used. One-way ANOVA with least significant difference (LSD) post-test was used to evaluate the statistical significance. The palatal trauma mainly disturbed the transverse development of the maxilla. ADM promotes mucosa healing, but there is still an inhibitory effect on maxillofacial growth.

## 1. Introduction

The hard palate sits at the front of the roof of the mouth and contains the palatine bone, making up two-thirds of the palate, separating the oral cavity and nasal cavity, participating in feeding and speech [1]. Palatal tumor or fracture, cyst resection, or scar contracture after surgical repair can lead to palatal trauma [2,3,4]. In the pediatric population, the child’s propensity to place objects in their mouth, or sabotage physical accidents, along with their unsteady gait, makes palatal trauma more common. Meanwhile, patients with cleft palate who receive palatoplasty or the use of relaxation incision also suffer from palatal trauma caused by surgery [5]. Palatal trauma in the adult population affects their phonation and mastication. In children and adolescents at the peak of growth and development, besides the problems mentioned above, palatal trauma may also inhibit maxillofacial growth [6,7].

This core concept, that palatal trauma has an inhibitory impact on maxillofacial growth, is clear [8]. Previous studies proved that palatal trauma had an adverse impact on maxillary growth [9]. However, what traumatic types and degrees of trauma contribute to the specific inhibition and how they affect the growth quantitatively still needs further investigation. The exact correlation of the trauma to the maxillary inhibition has not been demonstrated. Additionally, there was no quantitative analysis of maxillary growth in longitudinal human studies based on this concept as we cannot control the trauma in humans; what we can do is to avoid the trauma as much as possible. In the animal study, we can control the trauma as one single factor and evaluate the maxillary growth comprehensively.

Another challenge is how to manage the wound healing regarding palatal trauma. Acellular dermal matrix (ADM) may be a practical and effective method for palatal wounds because it solves the problems of insufficient tissue and scar formation that are unavoidable with most surgical procedures [10]. ADM is a soft connective tissue graft generated by a decellularization process that preserves the intact extracellular skin matrix. Upon implantation, this structure serves as a scaffold for donor-side cells to facilitate subsequent incorporation and revascularization [11]. ADM for the treatment of palatal fistula was first reported by Kirschner in 2006 [12]. In large animal models with palatal trauma, ADM has a good curative effect during the palatal wound healing process, as no scar formation was observed [10]. As we mentioned before, different types of palatal trauma may inhibit the maxillofacial growth; while the wound healing process can be assisted by ADM application; whether ADM can alleviate the inhibitory impact also needs to be confirmed.

In this study, we mimicked different types and degrees of palatal trauma on a young rat model, including soft and hard tissue trauma [8,13], and ADM was also applied to cover the wound. Our experimental comparative study aimed to determine the influence of different degrees of palatal trauma on maxillofacial growth and assess whether usage of ADM can help rescue the inhibited growth during palatal wound healing. This study would help the surgeons comprehensively understand the impact of palatal trauma on maxillary growth and the therapeutic effect of the ADM.

## 2. Materials and Methods

### 2.1. Animal Model Establishment

Three-week-old Sprague Dawley rats (male, 55 ± 5 g), which were considered to be infant rats, were purchased from the Dashuo Experimental Animals Company (Sichuan, China) and all animals were housed in pathogen-free conditions. Six groups were included in this study. For Group I to III and VI to V, there were 15 rats per group; for group VI, there were 25 rats. Animals were fasted for 8 h before operations and fixed after intraperitoneal anesthesia (10% chloral hydrate, 3.5 mL/100 g). For disinfection in and around the mouth, 1% iodophor was used, and 75% alcohol was used for deiodination. The mouth of the rat was opened by a gag. Gentamicin was given three days after the procedure. Adequate measures were taken to minimize the pain or discomfort of the animals. The design of animal experiments was approved by the Ethics Committee of West China Hospital of Stomatology (Sichuan, China).

### 2.2. Various Degrees of Palatal Traumas

Various traumas were mimicked on the hard palate of rats. Necessary analgesic medication was used for pain control after the operation.

**Figure 1:**
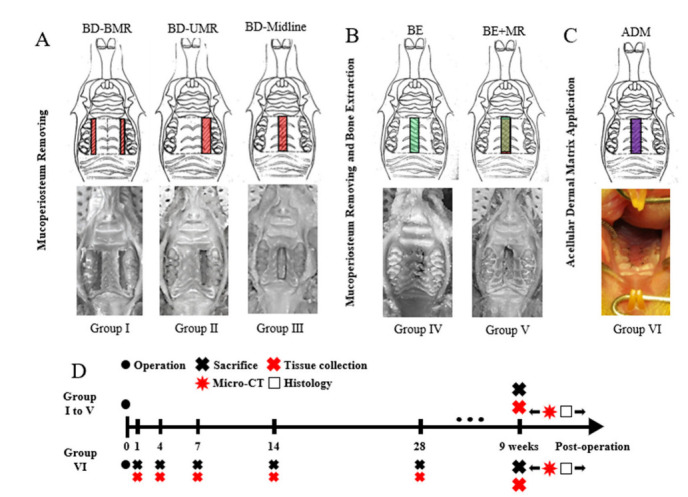
Various degrees of trauma on the hard palate and ADM-treated model. (**A**) Three types of mucoperiosteum removal on the hard palate led to bone denudation (BD). Group I: Bone denudation caused by bilateral mucoperiosteum removal (BMR). Group II: Bone denudation caused by unilateral mucoperiosteum removal (UMR). Group III: Bone denudation caused by mucoperiosteum removal on the midline of the hard palate. (**B**) Two types of bone extraction (BE) on the hard palate. Group IV: Bone extraction (BE). Group V: Bone extraction and mucoperiosteum removal (BE + MR). (**C**) Application of acellular dermal matrix (ADM) to cover the bone denudation. (**D**) Experimental schedule of the animal study: For Group I to V, the rats were sacrificed nine weeks after the operation; for Group VI, rats were sacrificed on Day 1, Day 4, Day 7, Day 14, and Day 28 (2 rats per time), and rest of the rats were also sacrificed nine weeks after the operation.

Firstly, three different locations of bone denudation (BD) by removing mucoperiosteum on the palate were manipulated (Figure 1A), including: Group I, BD caused by bilateral mucoperiosteum removal (BMR) on bilateral molar areas; Group II, BD caused by unilateral mucoperiosteum removal (BD-UMR) on the unilateral molar area; Group III, BD in the midline of the palate (BD-midline). For these three groups, the entire layer of mucoperiosteum was incised and removed to expose the bone surface. The total area of BD in four groups was kept at 1.5 mm × 4 mm (For BD-BMR, 0.75 mm × 4 mm of BD was set on both sides). For experiments on bone defects on the hard palate, two groups were designed as follows: Group IV, Bone extraction (BE) (1.5 mm × 4 mm) on the midline of the palate without mucoperiosteum removal; Group V, BE (1.5 mm × 4 mm) on the midline with MR (BE + MR) (Figure 1B). Observing the mucosa of the nasal surface was the standard for a successful BE. In the group VI, we added the animal model with the acellular dermal matrix (ADM) (ZH-BIO, Yantai, China) to cover bone denudation (Figure 1C). Mucoperiosteum (1.5 mm × 4 mm) was firstly removed, followed by suturing the ADM without tension to cover the bone denudation. For observing the tissue healing process, an extra six rats were needed for sample harvesting of six-time points.

### 2.3. Sample Harvesting

For each group except the ADM group, the rats were sacrificed by cervical dislocation nine weeks after the operation. For the ADM group, rats were sacrificed on Day 1, Day 4, Day 7, Day 14, and Day 28 (2 rats per time) to observe the tissue healing. The remaining 15 rats of the ADM group were also sacrificed nine weeks after the operation (Figure 1D). The heads of rats were collected with mandible removal. Soft tissues on the bone surface in samples were removed, and samples were fixed in 4% paraformaldehyde at 4 °C overnight. The fixed samples were rinsed with tap water for 6 h and then stored in 70% ethanol at 4 °C for further testing.

### 2.4. Micro-CT Scanning and Measurement of the Maxillofacial Growth

**Figure 2:**
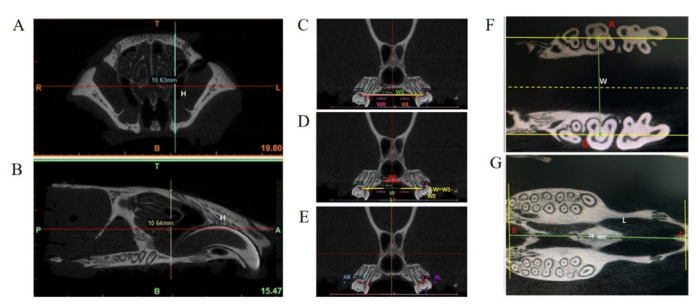
Parameters to characterize the maxillofacial growth. (**A**,**B**) The maximum perpendicular distance on the coronal and sagittal planes of the front maxillary suture to the alveolar crest was defined as the height of the maxilla (H). (**C**) Dental arch width (W1) was defined as the distance between the midpoints of the line connecting buccal and palatal CEJs of the teeth on both sides, and use the intersection of this line and central axis (crossing cranial fissure, nasal septum, and palatine fissure) as the separation to measure the right and left dental arch width (WR and WL). (**D**) The palatine bone width on the layer (W2) was measured to demonstrate the horizontal distance of the great palatine neurovascular bundles on both sides; alveolar bone width (ΔW = W1−W2) was the difference between the dental arch width and palatine bone width, which could represent the growth of alveolar bone. (**E**) The right and left inclination angles of the dental arch (AR° and AL°) were the intersection angle of the occlusion plane and the long axis of the tooth. (**F**) On the layer that showed the cementoenamel junction (CEJ), the distance between the midpoints of the buccal and palatal surface of the second molar was defined as the width of the maxilla (W). (**G**) The perpendicular distance between the anterior point of incisive foramen to the line connecting two posterior points of the distal root of the third molars was defined as the length of the maxilla (L).

The fixed samples were firstly used for micro-CT scanning (vivaCT80; SCANCO Medical, Brüttisellen, Switzerland). The scanning was performed at 145 mA and 55 kVp every 18 µm at high resolution. The width and length of the maxilla were measured with the SCANCO post-processing workstation (Figure 2) and defined as follows: On the layer that showed cementoenamel junction (CEJ), the distance between midpoints of the buccal and palatal surface of the second molar was defined as maxilla width (W); the perpendicular distance between the anterior point of incisive foramen to the line connecting two posterior points of the distal root of the third molars was defined as maxilla length (L). W and L (mm) were recorded as means of measurements on the near three layers (measured 3 times per layer).

The other parameters were measured by Mimics 16.0 (Materialise; Leuven, Belgium). The maximum perpendicular distance on coronal and sagittal planes of the front maxillary suture to the alveolar crest was defined as the maxilla height (H). The following parameters were measured on the coronal layer that contained distobuccal and distopalatal roots [14]: (1) Dental arch width (W1) was defined as the distance between midpoints of the line connecting buccal and palatal CEJs of teeth on both sides, and the intersection of this line and central axis (crossing the horizontal midpoint of the cranium, nasal septum, and the horizontal midpoint of the palatine bone) was used as separation to measure the right and left dental arch width (WR and WL); (2) the palatine bone width on layer (W2) was measured to demonstrate the horizontal distance of greater palatine neurovascular bundles (PNBs) on both sides; (3) alveolar bone width (ΔW = W1−W2) was the difference between dental arch width and palatine bone width, which could represent alveolar bone growth; (4) the right and left inclination angles of the dental arch (AR° and AL°) were the intersection angle of occlusion plane and the long axis of the tooth. The parameters were measured at least 3 times, and the measurements were required to be repeated after two-week intervals by the same investigator. The means of measurements were recorded.

### 2.5. Histological Analysis of the Palate

After the micro-CT scanning, the samples were decalcified in 10% ethylene diamine tetraacetic acid-PBS solution at 4 °C for one month. The decalcified maxilla and the fixed tissues were dehydrated, embedded in paraffin wax, and sectioned (4 µm). The hematoxylin-eosin (HE) stain and Sirius Red stain were performed using a staining kit (both were purchased from Beijing Solaibao Technology Co. Ltd., Beijing, China) according to the manufacturer’s instructions.

### 2.6. Statistical Analyses

All numerical data were expressed as means ± standard deviation. One-way ANOVA with the least significant difference (LSD) post-test was used to evaluate the statistical significance (* *p* < 0.05, ** *p* < 0.01, and *** *p* < 0.001). Statistical analyses were performed using SPSS 22.0 statistical software (SPSS Inc., Chicago, IL, USA).

## 3. Results

### 3.1. The Impact of Different Degrees of Palatal Trauma on the Maxillofacial Growth

**Figure 3:**
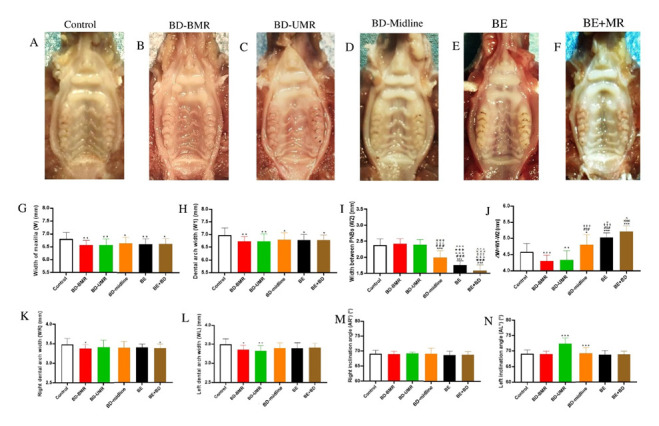
(**A**–**F**) The palatal rugae and scar on the palates at nine weeks after various degrees of palatal trauma. (**G**–**N**) Parameters demonstrated the inhibitions of the maxillofacial growth after bone denudation and destruction. One-way ANOVA was used to evaluate the statistical significance (* *p* < 0.05, ** *p* < 0.01, and *** *p* < 0.001). *, compared with the control group; #: compared with the BD-BMR group; +: compared with the BD-UMR group; × compared with the BD-midline group; ˄: compared with BE-group.

The injured palates were healed and covered by mucosa in different groups at nine weeks postoperatively. Differences can be found in the palatal rugae in the experimental groups compared to the control group, and deformities are mainly correlated to traumatic locations (Figure 3A–F). Scarring could also be found along with the lesion’s original location (Figure 3B–F). When visualized under polarized microscopy, Sirius Red (SR)-staining outcomes of BD-BMA and BD-UMA groups show that the appearance of Sharpey’s fiber on molar areas may restrict the width of the maxilla whereas BD-Midline group is different (Figure S1) (Beijing Solaibao Technology Co. Ltd., Beijing, China).

#### 3.1.1. Width of the Maxilla (W) and Dental Arch Width (W1)

W and W1 of the different experimental groups were all significantly decreased compared to the control group (Figure 3G–H). However, there is no statistical significance among thetically different degrees of palatal trauma groups. (*p* < 0.05).

#### 3.1.2. Width between PNBs (W2) and ΔW = W1−W2

As the degree of palatal injury increased, the width between PNBs (W2) in BD-midline, BE, and BE + BD groups gradually decreased and was shorter than that in the control group (*p* < 0.001) (Figure 3I). By contrast, the ΔW was gradually increased in the BD-midline, BE, and BE + BD groups and was wider than that in the control group (*p* < 0.001) (Figure 3J). However, ΔW in the BD-BMR and BD-UMR was shorter than that in the control group.

#### 3.1.3. Right Dental Arch Width (WR), Left Dental Arch Width (WL), Right Inclination Angle (AR°), and Left Inclination Angle (AL°)

The right dental arch width in both the trauma group of BD-BMR and BE + BD were shorter than that of the control group (*p* < 0.05) (Figure 3K). The palatal trauma caused by BMR and UMR inhibited the left dental arch width (*p* < 0.05) (Figure 3L). As for the right inclination angle, there is no significant difference among the different groups (*p* > 0.05) (Figure 3M). The left inclination angle in BD-UMR group and BD-midline group was wider than that of the control group (Figure 3N).

### 3.2. The Application of ADM to Treat the Bone Denudation Caused by Mucoperiosteum Removal on the Midline of the Palate

**Figure 4:**
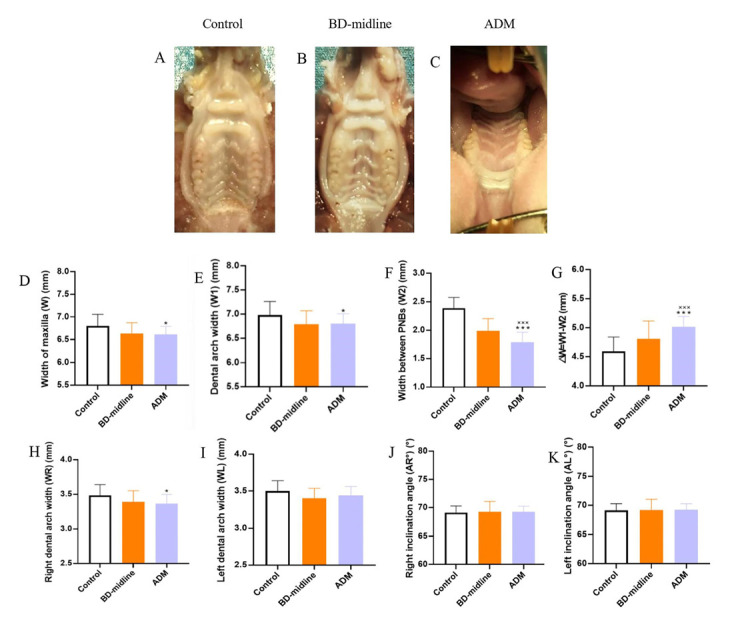
Inhibitions of the maxillofacial growth still happened after covering the bone denudation by ADM. Parameters demonstrated the inhibitions of the maxillofacial growth still happened after covering the bone denudation by ADM. One-way ANOVA was used to evaluate the statistical significance (* *p* < 0.05, *** *p* < 0.001). *: compared with the control group; ×: compared with the BD-midline group; (**A**–**C**) The palatal rugae and scar on the palates at nine weeks among three experimental groups. (**D**–**K**) Parameters demonstrated the inhibitions of the maxillofacial growth among the control group, BD-midline, and ADM group.

**Figure 5:**
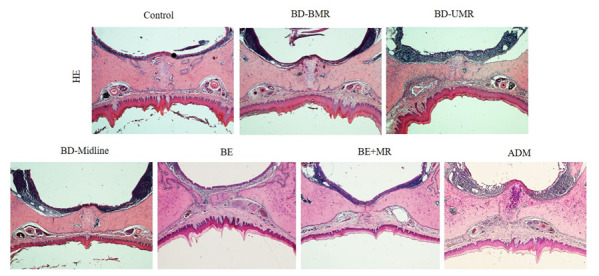
The representative histological finding with hematoxylin-eosin (HE) stains of the palate after various degrees of the palatal wound and ADM treated palatal wound nine weeks after surgery. Compared with the control, BD-BMR, BD-UMR, BD-midline, BE, and BE + MR, the thickness of the mucoperiosteum in the trauma area increased and the regenerated soft tissue is full of new small blood vessels. However, the periosteum thickness was almost not observed at the surgical site.

The comparisons of maxillary measurement data was conducted among the control group, BD-midline, and ADM group (Figure 4A–C). W, W1, W2, and WR in the ADM group were shorter than those of the control group (*p* < 0.001) (Figure 4D–H). In contrast, ΔW was wider in the ADM group compared with the other two groups (Figure 4G). There were non-significant differences in the left dental arch width, right inclination angle, and left inclination angle (Figure 4I–K). Compared with the control group and the bone denudation on the midline of the palate, the soft tissues were thicker in the ADM group, but the periosteum cannot regenerate (Figure 5).

**Figure 6:**
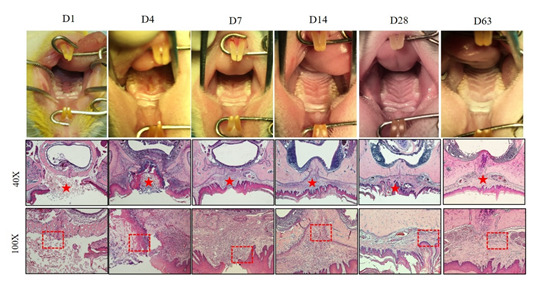
The postoperative recovery process and the representation of histological findings stain in the group using ADM to cover the bone denudation. In the ADM group, no noticeable scar was found on the palate nine weeks postoperatively. The healing process was finished around Day 28. Seven days after surgery, granulation tissue formation and angiogenesis were found between materials gaps, and epithelial cells migrated on the surface of ADM. Fourteen days after surgery, epithelial cells migrated on the surface of ADM, and blood vessels were reduced. Twenty-eight days after surgery, collagen fibers are arranged horizontally. The red stars represent the location of ADM; the red rectangles represent the junction of material and normal tissue junction.

During the recovery process in the ADM groups, the histological analysis has shown that mild infiltration of local inflammatory cells happened on the fourth day after the material implantation. ADM degradation following granulation tissue can be found on the seventh day. The horizontal collagenous fibers are formed until the twenty-eighth day postoperatively (Figure 6).

## 4. Discussion

The intact palatal soft and hard tissues contribute to stable and expected maxillary morphology and normal growth [13]. For those young patients who are in the bone development period, various kinds of trauma or surgical intervention can affect their maxillary growth [15,16]. Palatal trauma often happens during traumatism and operation [17].

Mucoperiosteum injury often occurred after periodontal flap procedure and palatoplasty. Laceration and puncture palatal wounds caused by sharp toys when children accidentally fall were ubiquitous in children. Hard palatal defects were common after maxillary fracture, the resection of palatal cyst, and tumor. These two types of palatal trauma would cause maxillary dental arch stenosis and malocclusion by the end of puberty. However, it is difficult to eliminate the potential confounding factors in the clinical features and to figure out the certain factor leading to maxillary deformity. Therefore, creating an animal model allowed for removing many of the confounding variables that were difficult to control in human studies and were fraught with interesting challenges.

A previous study suggested three factors that impact the normal development of local palatal bone, including nutrition of nerves, distribution of blood vessels, and mechanical force which were relevant to the normal development of local palatal bone. Different sizes and locations of denuded hard palates or different types of tissue trauma may change these factors to destroy the centers of ossification [14]. In this study, we simulated five degrees of palatal trauma in a young rat model to figure out its impact on maxillary growth. Then, the ADM was used to cure the palatal trauma to observe its therapeutic effect on mucoperiosteal healing and to figure out whether it can alleviate the inhibitory impact of palatal trauma on maxillary growth.

Firstly, the ΔW in the BD-BMR and BD-UMR groups was shorter than that in the control group. This is because the inhibition occurred in the posterior teeth where the mucoperiosteum was removed in these two groups. Secondly, as the severity of the palatal trauma that occurred in the midline increased, the palatine bone width (W2), which is the horizontal distance of the great palatine neurovascular bundles, became shorter and shorter. At the same time, the ΔW in the BD-midline, BE, and BE + BD groups (the palatal trauma that occurred in the midline) showed an increasing trend and was wider than the control group. This can be explained by the fact that the mucoperiosteum on the lateral side was undisrupted and the inhibition of the maxillary width was partially mitigated by a compensatory increase in bone formation in this position. Therefore, the phenomenon explained why in all five experimental groups, the growth of maxillary width and dental arch width was inhibited either by mucoperiosteum removal or bone destruction in the palate, but there was no difference among different palatal trauma groups. Wijdeveld MG [17] performed palatal surgery on beagle dogs and concluded that palatal surgery mainly disturbed the transverse development of the maxillary dental arch, especially when the surgery was performed before or during the transition of the posterior teeth. Some scholars concluded that the narrow dental arch and horizontal growth inhibition of the maxilla could correlate to the continuous tension caused by collagen fibers of the scar tissue [18]. We think that maxillary growth is mainly closely related to the osteogenesis of mucoperiosteum and the inhibition of maxillary growth only occurred locally where the mucoperiosteum was removed. When the trauma occurred close to the molar areas but not the midline, Sharpey’s fiber would form. It is unclear whether the formation of Sharpy’s fiber is related to an increase in the degree of teeth inclination and the inhibition of maxillary development.

Acellular dermal matrix (ADM) has been described as an adjunct in primary cleft palate repair to reduce the fistula rate in several retrospective studies [14,19,20]. In our research, we further estimated the role of ADM in treating bone denudation and alleviating inhibition of maxillary growth. From the histological analysis, the process of regeneration took at least twenty-eight days which is the same as the normal physiological processes [21]. However, the number of collagen fibers increased, and no evident regeneration at the lesion was found in the ADM-treated group. However, the growth inhibition still happened after the denudation area was covered by ADM, and the inhibition became even more severe in the midline area. The possible explanation could be that ADM blocked the migration of the tissues from the lateral side to the midline, and ADM cannot promote mucoperiosteum regeneration. The outcomes were similar to those found in former studies, that the dermal substitute would not alleviate the growth inhibition [19,21].

Collectively, our study simulates different severity degrees of palatal trauma that may occur in our daily life, particularly mucoperiosteal injury and discontinuous bone. Specifically, the inhibition development of local bone covered by damaged mucoperiosteum is the reason for the inhibition development of the whole maxilla. Meanwhile, other bone areas covered by mucoperiosteum still have a great ability for osteogenesis. ADM plays a major role in the regeneration of muscle [22], connective tissue [23], and skin tissue, but not in the bone and mucoperiosteum. We should avoid unnecessary surgical maneuvers when doing surgery and decrease the risk of surgical trauma on the mucoperiosteum. However, there is still some lack in our study. A more specific molecular biological study should be carried out to figure out the osteogenic mechanism of mucoperiosteum. Meanwhile, the recapitulations of palatal trauma in this study are still idealized because the trauma under real situations cannot be well-defined. Thus, retrospective studies are still needed.

## 5. Conclusions

**Figure 7:**
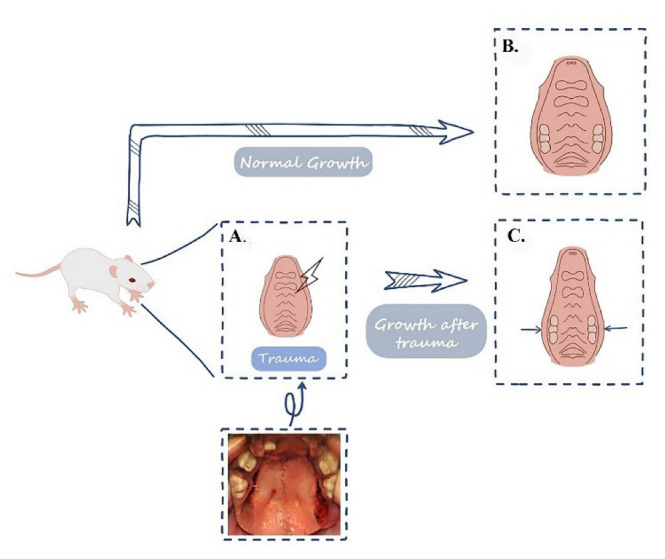
Palatal trauma inhibits the maxillary width. (**A**) Palatal trauma, such as relaxing incision on the hard palate, was created in the palate of the rat. (**B**) The normal growth of the palatal of the rat. (**C**) The development of the maxilla is inhibited.

It is very important to promote transverse maxillary growth by maintaining the palatal mucoperiosteum integrity and bone continuity. If the palate was partly damaged, the undisturbed position can take the role of compensatory growth to maintain the maxillary width (Figure 7). Although ADM contributes to facilitating tissue repair or regeneration, it cannot substitute the role of mucoperiosteum in promoting osteogenesis. Therefore, intraoperative procedures should be refined to avoid damage to the mucoperiosteum, especially in cleft palate repair, periodontal surgery, and palatal fracture repair.

## Data Availability

The data that support the findings of this study are available from the corresponding author, [H.H.Y.], upon reasonable request.

## Supplementary Materials

The following supporting information can be downloaded at: https://www.mdpi.com/article/10.3390/biology11081142/s1, Figure S1: Representative Sirius Red stain in palate tissues at nine weeks after bone denudation.
