# Supplementary material for: Observation of Palatal Wound Healing Process Following Various Degrees of Mucoperiosteal and Bone Trauma in a Young Rat Model
Source: Biology (Basel). 2022 Jul 29;11(8):1142. doi: 10.3390/biology11081142 (PMC9405411; doi:10.3390/biology11081142)
Supplement: Supplementary file 1 [file biology-11-01142-s001.zip › biology-1814111-supplementary.pdf]

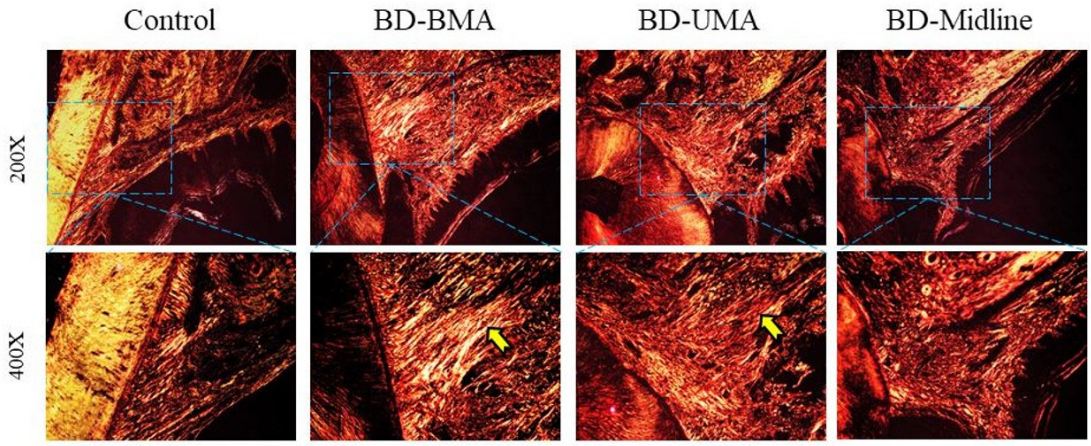

Figure S1. Representative Sirius Red stain in palate tissues at nine weeks after bone denudation.

Sharpey's fiber could be found to connect the palatine bone and the tooth neck in BD-BMA and BD-UMA groups. Yellow arrows showed Sharpey's fiber.
